# Substance of Lectures on Gold

**Published:** 1878-03

**Authors:** 


### ARTICLE III.

### *Substance of Lectures on Gold.*

BY PROF. HODGKIN.

It is not a little singular that it is almost to the dentist alone that gold has any intrinsic value. True, it is used by painters and gilders, but even with them it is used only in ornamentation. As a circulating medium and as an article of personal adornment it is very largely used, but we will understand that ornaments have no intrinsic worth, and that for the purposes for which gold coin is used, any other metal would answer, did mankind but agree upon it as a common medium having a common value. To the artisan, for industrial purposes, a bit of iron or steel is vastly more useful—has greater intrinsic worth—is more valuable in itself than its equivalent weight of the precious metal, which for us so largely represents our material comfort. For gold means food, clothing, shelter, the comforts of life, the means of transportation,—in a word all that we include in living, except perhaps air, and in some cases, water.

To the dentist it is valuable not only for the purposes of life or comfort, but it has an intrinsic value—*i. e.*—a value in itself, largely used in his work, as iron or brass to the artificer, as wool or cotton to the weaver, and so on. That this value is not so fully recognized in this our day of dentistry, (I might call it the degenerate day of dentistry, at least in the application of gold to dental uses,) as formerly, is a fact I am painfully familiar with; and I shall feel that it is at once a duty as well as a privilege to call your attention to some of its former uses in our profession, in the hope that its value may again be recognized, and it resume the position in dental art it rightfully claims, and which for many years it held undisputed possession.

As a medium of circulation gold is one of the oldest of the discovered metals. The earliest recorded history of the earth—the Bible—in its earlier chapters, shows us that in the primitive days of our ancestors they had discovered and made use of for personal ornamentation, this precious metal. And it seems to be understood that its use as a circulating medium was began nearly as early. Although by no means the most rare of the metals, it has held its place as the most valuable,—that is of those that are commonly known—to the present day.

It is a wonderful and curious study to us, in this materialistic day, to revert to the earlier days of chemistry, and ponder over the whimsical notions of the alchemists, those weird and mysterious sages, who in secret consult with nature, delved out so many of her closely locked secrets, and whose labors, obscure, absurd, and fantastical as they seem to our enlightened understandings, were notwithstanding, all-important to us, as laying the foundation of our modern physics; labors whose very obscurity and mystery gave their performers a distinction and eminence peculiarly their own, in the days when to be mystical was to be awed, and when all who found treasures of intellect hoarded them as closely as the miser hoards the "Aurum" of which we speak.

A blessed day, this of ours, when it is no longer the province of science to conceal, but to divulge, when no secret of nature is safe with its discoverer, but is the common property of the world of science. It is only of late, very late as to dentistry, that this is true, and we may thank our stars for living now, instead of when the dentists' secret lived and died with him, and when his laboratory was as carefully guarded as that of Paracelsus.

One of the earliest notions to be originated, and one of the most difficult it would seem to eradicate, was that of transmuration—that gold was convertible from other metals; and innumerable were the attempts at finding the "touch-stone," whose magic presence should cause the valuable yellow metal to come forth from base mercury or other substances.

The law of compensation, which ordains that even the efforts of selfishness shall result in good, evolved much that is useful in our modern chemistry out of these crude and abortive attempts at finding a magical something by whose agency transmutation could be effected. But another and higher law seemed not thus early recognized, and that is that the truly valuable, in the sense in which gold is valuable, must be comparatively scarce, and that to increase its amount indefinitely, would be to cause it to lose that value; and the failure further to recognize the fact, which we as dentists ignore too constantly and habitually, that the truly valuable must be costly,—must result from effort, labor, toil of brain and body. It is a law of the human mind, as universal and unchangeable as eternity, that men do not value that which costs them little. See, then, that you give your patients the best work; see also that they adequately recompense you for your efforts.

But I am spending too much time in these speculations and reflections, and must pass on to that which is more immediately before us. We are all probably aware that gold is usually intimately associated with quartz, and that even the gold found in the beds of the rivers and mountain streams was washed down by these streams from its quartz bed, and is accompanied by and associated with sand, which is simply pulverized quartz. But a fact we may not be so familiar with is that the gold ore from the different gold-bearing districts is uniformly about the same in purity and in association with other metals. For example the gold from our own California ranges in purity from 865 to 890 per 1000 and the alloying metal is principally silver; that from Australia ranges from 933 to 982 per 1000; Russian gold is associated with iron, platinum and palladium; while the ore from Brazil is alloyed with silver and copper. Wherever we find gold we may pretty generally know its associated metals by the knowledge of the country in which it is discovered, for, as we have seen, its alloying metals are constant in the same region.

I am reminded also of another fact of importance to the refiner and assayer, and that is that gold, although found associated and alloyed with these inferior metals,—and I have only mentioned a few—is always gold in a metallic form, that is to say we never find the sulphate or chloride, &c. of gold. It is a royal metal, and although by stress of circumstance, or by the potent agency of heat, it may be melted and alloyed with these others, it proudly refuses to obey the chemical laws and affinities which have their free play amongst its inferiors, and preserves its identity amid all changes.

The question of the elimination of the baser metals from the gold and the obtaining it in a state of purity, is one that must engage our attention further on, and for the present we will only say that the "behavior" of the gold is materially influenced by the presence in ever so small quantity of most of the metals, and notably so by some of the baser sort.

As we see it, there seems to be but little gold. Have other nations more than we? Not as currency I mean, but as a product of national industry. Out of the bowels of the earth are brought to light and utilized invaluable stores, among these gold. As to the total production, we are informed that the whole product of the world for 1865 was 559,587 pounds, nearly two hundred and eighty tons! We would hardly have thought that, considering our own needs and poverty. Enough to load over twenty cars, and a hard pull for at least one locomotive! California gave us in eighteen years of her most prolific yield, eight hundred and thirty-six millions of dollars worth of the precious metal, while Australia in ten years yielded five hundred and seventeen milllions.

But we tire of these sums of nine figures, purely theoretical or hypothetical to us, for we wish to see something of the methods by which the gold is obtained from its quartz matrix, or its sandy river-bed.

Two properties of gold are made use of here as available in simplifying this task of separation. First the specific gravity of gold, and second, its affinity for mercury: with these two properties, and with some very simple apparatus, we are prepared for the work of gathering the shining grains which are scattered through the sand, or mingled with the quartz which we have by suitable crushing-mills, pulverized. In passing, however, it is curious and interesting to relate that gold is found not only in grains and scales, as in the sands of the streams flowing through auriferous districts, but is found in veins, and also in pieces called nuggets. Of these the largest that I have record of was found in Australia and weighed two hundred and thirty-three pounds! It was called the "Sarah Sands," whether because a female of that name was the lucky finder, or whether the male finder classed it in value with his sweetheart of that name,

I have not the least idea. To find fifty-five thousand dollars in a chunk was certainly, if there be such a thing, luck. These nuggets are of surprising purity, as a mass of eighty-four pounds weight, only lost a few grains in melting,—it was practically pure.

The specific gravity of gold is very great—more so than of any other substance with which we are acquainted, except Platinum. It is nineteen and a-half times heavier than water. We can best form an idea of its weight by comparison with, for example, lead, which we are in the habit of proverbially calling heavy. Now the specific gravity of lead is only eleven, or in other words it is about eleven times as heavy as water, while gold is nearly twenty times as heavy. So that we see that gold is nearly twice as heavy as lead. Advantage is taken of this weight to separate the gold from the sand in what are called the "washings." A simple inclined plane—a board set at a gentle slope, with sides, making a shallow trough, and strips of wood across the bottom about a foot apart, constitutes the simple apparatus used by the miners, at least in the more primitive methods of work. Water is an indispensable element in this process, and times of drought are times of inactivity among the gold seekers. The apparatus is usually placed in the flowing stream, and the sand, mingled with water, is poured in at the upper or elevated end. The gold sinks and is caught on the cross strips, while the sand is washed out, carrying with it so much gold that it usually pays to go over these washings by a second and more complicated process.

Such in brief is gold washing, the principle being the same in all the various machines made for the purpose. We have yet to examine the process involving the other property spoken of—that of the affinity of gold for mercury.

It would be difficult to persuade any one that mercury is capable of being made solid, unless they had faith in the statements of the few who have seen it. But although none of us probably have seen this, yet we are satisfied that such a thing is possible. But curious and difficult of acceptation as is this fact, a still more curious one is that mercury is capable of making gold fluid like itself, by combination with it. These alloys,—for such they seem to be,—of gold and other metals with mercury, are called amalgams, and there are few metals with which mercury will not amalgamate. Iron is one of the few. With gold it unites readily in all proportions, the only requisite being that the two metals shall be brought into intimate contact. The gold, intermingled with the sand and other impurities, is brought in contact with mercury, and as the property of the last, as I have said, is to render the other fluid, you will see how valuable an aid the mercury is in the accumulation and gathering together of the dust and grains of gold which on account of their minuteness would otherwise be lost. The amalgamation is facilitated greatly by sodium, and in this way much of the refuse of the first workings is profitably rehandled. The mercury is readily separable from the gold by distillation, and by subsequent condensation is used over again.

Lest any of you should be captivated by the statements concerning this precious metal, and feel inclined to rush off to the Black Hills or other new-found gold territory, I assure you that of all the precarious and hazardous methods of making a fortune this is the most precarious and hazardous, and at the same time the most laborious. The Spaniards have a proverb that a gold mine yields lead, a silver mine yields copper, a copper mine yields gold; meaning thereby that the most profitable mining is copper, the next silver, and the least of all, gold. And the experience of miners seems to substantially prove this. Even should one be so lucky as to strike "pay dirt," amid that wild and reckless crowd which is allured to such places by the enticing seductions of the auriferous metal, the chances are that he would be knocked on the head by his less fortunate comrades, and his discovery prove the most costly of his life. With your excavators in dentine you will be more likely to find gold, or at least the means of getting it.

